# Facial Fabrication of Large-Scale SERS-Active Substrate Based on Self-Assembled Monolayer of Silver Nanoparticles on CTAB-Modified Silicon for Analytical Applications

**DOI:** 10.3390/nano11123250

**Published:** 2021-11-30

**Authors:** Juanjuan Guo, Yang Xu, Caili Fu, Longhua Guo

**Affiliations:** 1 College of Oceanology and Food Sciences, Quanzhou Normal University, Quanzhou 362000, China; gjjfst15@163.com; 2 College of Physics & Information Engineering, Quanzhou Normal University, Quanzhou 362000, China; xu_yang1990@126.com; 3 National University of Singapore (Suzhou) Research Institute, No. 377 Linquan Street, Suzhou Industrial Park, Suzhou 215128, China; caili.fu@nusri.cn; 4 College of Biological, Chemical Sciences and Engineering, Jiaxing University, Jiaxing 314001, China

**Keywords:** surface-enhanced Raman spectroscopy (SERS), SERS-active substrate, self-assembled monolayer, silver nanoparticles, enhancement factor

## Abstract

Surface-enhanced Raman spectroscopy (SERS) has been proven to be a promising analytical technique with sensitivity at the single-molecule level. However, one of the key problems preventing its real-world application lies in the great challenges that are encountered in the preparation of large-scale, reproducible, and highly sensitive SERS-active substrates. In this work, a new strategy is developed to fabricate an Ag collide SERS substrate by using cetyltrimethylammonium bromide (CTAB) as a connection agent. The developed SERS substrate can be developed on a large scale and is highly efficient, and it has high-density “hot spots” that enhance the yield enormously. We employed 4-methylbenzenethiol(4-MBT) as the SERS probe due to the strong Ag–S linkage. The SERS enhancement factor (EF) was calculated to be ~2.6 × 10^6^. The efficacy of the proposed substrate is demonstrated for the detection of malachite green (MG) as an example. The limit of detection (LOD) for the MG assay is brought down to 1.0 × 10^−11^ M, and the relative standard deviation (RSD) for the intensity of the main Raman vibration modes (1620, 1038 cm^−1^) is less than 20%.

## 1. Introduction

Since the discovery of surface-enhanced Raman scattering (SERS) in the 1970s [1,2], it has exhibited extraordinary potential in different fields due to its inherently high sensitivity. The early SERS substrates were normally prepared by roughening a flat Au or Ag substrate using oxidation–reduction cycles [3]. However, researchers found that SERS substrates fabricated using this method usually have random nanostructured features with a wide size and shape distribution on their surface. Thanks to the development of nanoscience and nanotechnology, in the past two decades, diverse nanostructures with well-controlled morphologies and sizes were utilized for the fabrication of SERS substrates [4,5,6,7]. Previous studies have also demonstrated that nanogaps between two or more particles are closely related to local electromagnetic phenomena, which are extremely sensitive to SERS enhancement factors. For example, the SERS signal can increase by two orders of magnitude when the gap distance decreases from 35 to 10 nm [8]. Thus, the nanogaps with extremely high enhancement factors are referred to as SERS “hot spots” [9,10,11]. Based on these findings, in the past decade, there has been a lot of research focused on the fabrication of SERS “hot spots” that have an extremely high detection sensitivity. SERS “hot spots” with single-molecule detection sensitivity have also been reported [12,13,14,15,16].

As stated above, the sensitivities of SERS-based analytical approaches are now equal or even better than most of the state-of-the-art analytical methods, such as fluorescence- [17,18], mass spectrometer- [19,20], and chemiluminescence-based [21] approaches, whose sensitivities are usually in the range of nanomoles per liter. However, the other problem that prevents the real-world application of SERS-based analytical approaches lies in the relatively poor reproducibility and uniformity of SERS-active substrates, which usually perform poorly when practically applied to the detection of real samples [22,23]. Therefore, uniformity and stability are key constraints that seriously affect the practical application of SERS, regardless of how well they have been enhanced in laboratory settings. Therefore, a lot of resources have been devoted to fabricating large-scale SERS substrates that have high-density hot spots as well as good uniformity [24,25,26]. For example, anodic aluminum oxide (AAO) film is used as a template to fabricate large-scale, 3-dimentionally assembled Au nanobipyramid (Au NBP) films [27,28,29]. The fabricated Au NBPs-AAO substrate displays much better reproducibility as well as four-order enhanced sensitivity for the detection of DA compared to a conventional silicon wafer substrate. Gold nanoplates with an edge length of 134 ± 6 nm were used to fabricate large-scale gold nanoplate films with tunable localized surface plasmon resonance (LSPR) and surface-enhanced Raman scattering (SERS) activity [30]. The corresponding SERS enhancement factor is as high as 5.4 × 10^7^. As well as the fabrication of nanostructures on rigid substrates, the fabrication of flexible SERS substrates also demands the attention of the research community [31]. Our group fabricated flexible SERS substrates by decorating commercial tape with colloidal gold nanoparticles (Au NPs), which simultaneously provide SERS activity as well as the “stickiness” of an adhesive. The utility of SERS tape was demonstrated when it was used to directly extract pesticide residue from fruits and vegetables via a simple and viable “paste and peel off” approach [32]. Recently, nanoimprint lithography was also used to fabricate large-area flexible SERS substrates on polycarbonate (PC) sheets using thermal imprinting. Highly uniform SERS substrates can be developed using a wafer that has an area of ~6 in. as part of a batch process [33].

It should be noted that although the presence of robust, reliable, and large-scale SERS-active substrates has been reported, the advances in this area are barely enough to satisfy the requirements for real sample analysis. In this work, we describe a convenient and cost-effective method for fabricating highly ordered Ag nanoparticle (AgNP) arrays that exhibit reproducible and controllable SERS activity. A common and cheap surfactant, cetyltrimethylammonium bromide (CTAB), is used as the connection agent. A self-assembled bilayer of CTAB is firstly formed on the surface of a glass substrate using a head-by-head assembly. The negatively charged AgNPs are then self-assembled on the CTAB layer through electrostatic interaction. During the airing process, the distance between the self-assembly AgNPs decreases with the contraction of the CTAB, creating abundant “hot spots”.

## 2. Experimental Section

### 2.1. Materials

PVP with an average molecular weight of 40,000 (PVP K30), AgNO_3_ (purity > 99.8 wt %), PEG 600, 4-methylbenzenethiol (4-MBT), malachite green (MG), and CTAB were purchased from Sinopharm Chemical Reagent Co., Ltd. (Shanghai, China). Silicon wafer was purchased from the FuZhou Gao Te company (Fuzhou, China). All other reagents were of analytical grade and were used without further purification. Deionized water with a resistance of 18 MΩ cm was used throughout the study.

### 2.2. Instruments

The UV-visible absorption spectrum was measured using a UV/VIS Perkin Elmer Lambda750 (PerkinElmer ltd., Waltham, MA, USA). Raman spectra were recorded on a Renishaw in Via confocal Raman spectrometer using a 633 nm laser line as the excitation source. The spectra were obtained by using a 50× objective lens to focus the laser beam onto a 1 µm^2^ spot. All of the Raman spectra presented in this work were the result of a single 35 s accumulation. Scanning electron microscopy (SEM) were carried out using the Nova Nano SEM230 (Alfa Chemistry, New York, NY, USA), which was operated at an acceleration voltage of 5 kV. The transmission electron microscope (TEM) image was recorded using a HITACHI HT7700 (Hitachi, Ltd., Tokyo, Japan).

### 2.3. Synthesis of Silver Nanospheres

Silver nanoparticles (AgNPs) with an average diameter of 50 nm were prepared using the PEG-reduction method [34,35]. In detail, 444 mg of PVP was dissolved in 40 mL of PEG under vigorous stirring at 80 °C. The role of PVP in this reaction system is to protect the AgNP colloids from aggregation. Afterwards, 1 mL of freshly prepared AgNO_3_ (0.5 M) solution (acting as the precursor) was rapidly added into the above solution. The resulting solution was homogenized through vigorous stirring for 10 min and finally turned dark yellow. Subsequently, the resulting solution was transferred into a Teflon-lined stainless steel autoclave with a capacity of 80 mL. The autoclave was sealed and heated at 260 °C for 24 h, and was then allowed to cool to room temperature naturally. The AgNPs was collected via centrifugation and then washed several times with distilled water to remove the residual PEG and PVP.

### 2.4. Preparation of SERS Substrate

**Scheme 1:**
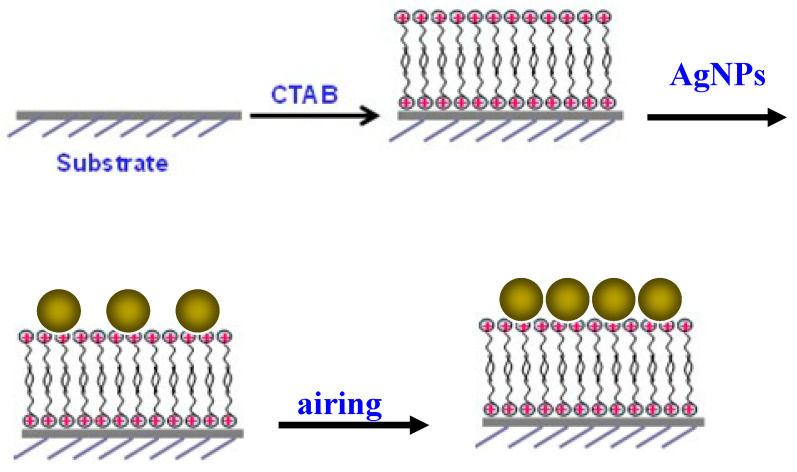
Illustration of the fabrication of substrate.

Scheme 1 depicts the procedure for synthetically producing the AgNP array-based substrates. Initially, the silicon wafer was immersed in a “piranha” solution (1:4 ratio of 30% H_2_O_2_ and concentrated H_2_SO_4_) for 15 min. Treatment with the “piranha” solution generated a dense monolayer of silanol groups on the surface of the silicon wafer. The silicon wafer was then immersed into a solution containing 0.05 M CTAB. In this process, a self-assembled bilayer of CTAB was formed on the surface via the head-by-head assembly. The CTAB-modified silicon wafer was then immersed into the AgNP colloid for 30 min. The negatively charged AgNPs self-assembled on the CTAB-modified silicon wafer through electrostatic interaction. Finally, the substrate was aired at room temperature.

### 2.5. Preparation of 4-MBT and MG Samples for SERS Measurements

In this process, 4-MBT (0.1 M) was prepared in ethanol, and MG was dissolved in distilled water. Firstly, an MG stock solution (1.0 × 10^−6^ M) was prepared in a 10 mL volumetric bottle. Then, the MG working solutions, with the concentrations of 1.0 × 10^−7^ M, 10^−8^ M, 10^−9^ M, 10^−10^ M, 10^−11^ M, and 10^−12^ M, were diluted with the distilled water one by one.

## 3. Results and Discussion

### 3.1. Characterization of AgNPs

**Figure 1:**
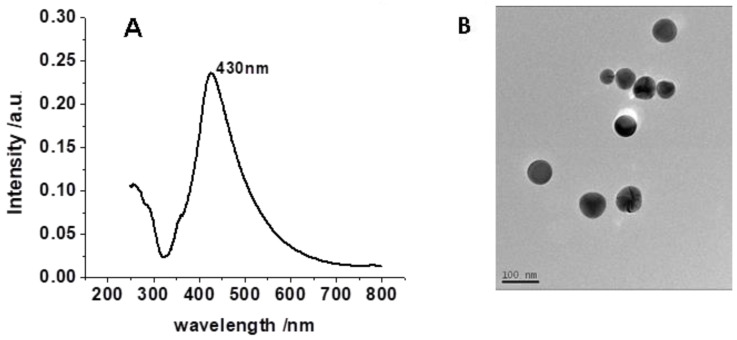
(**A**). The UV/vis extinction spectra of the AgNPs. The as-synthesized AgNP solution was used for this test. (**B**). TEM image of the AgNPs.

SERS is highly dependent on the morphologies and dimensions of the nanostructures [36]. It has been reported that the dimension of the optimized Ag nanospheres for SERS is about 50 nm [37,38]. As shown in Figure 1A, the prepared AgNPs possessed an extinction peak at 430 nm with a narrow full width at half-maximum, which is consistent with [34,35]. TEM images (Figure 1B) show that the prepared products are regular spherical particles with uniform size and without sharp or truncated corners. These nanospheres have smooth surfaces and a mean diameter of about 54 nm (with a standard deviation of 10 nm).

### 3.2. Morphologies of the Prepared Substrates

**Figure 2:**
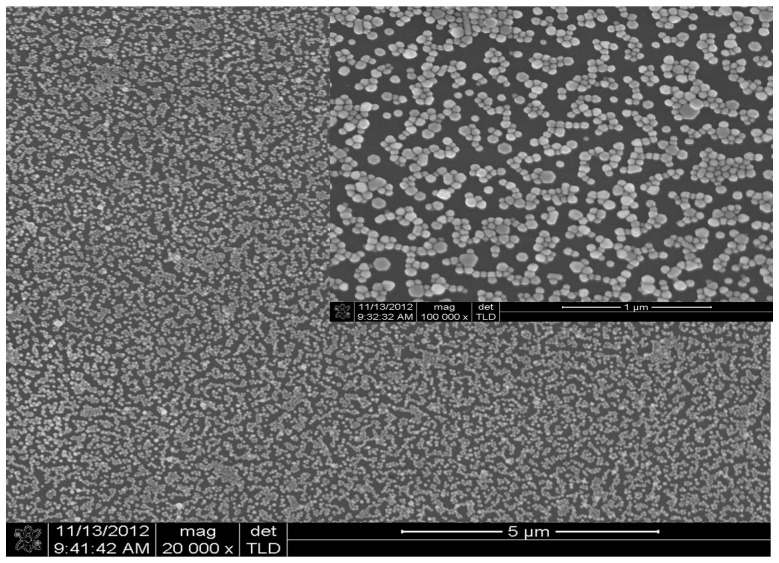
SEM images of prepared substrate.

Figure 2 shows the SEM images of the prepared AgNP array-based substrate. A more greatly magnified image of the substrate is provided in the inset. The high-resolution SEM image shows how the nanoparticles were formed through self-assembly. In this study, we introduce a convenient and efficient assembly method for fabricating SERS substrates. CTAB, a common and widely used surfactant, was used as the bifunctional ligand to link the solid substrate with AgNPs. As depicted in Scheme 1, during the airing process, the distance between the self-assembled AgNPs decreased as the CTAB shrank. The arrangement of the AgNPs on the silicon wafer is dense, generating a large number of “hot spots”. It is well known that, with the SERS effect, there is a large Raman enhancement in the “hot spots” of metallic nanostructures due to the strong local electromagnetic field coupling effect of the adjacent metallic nanostructures [39].

### 3.3. SERS Property of the Substrate

**Figure 3:**
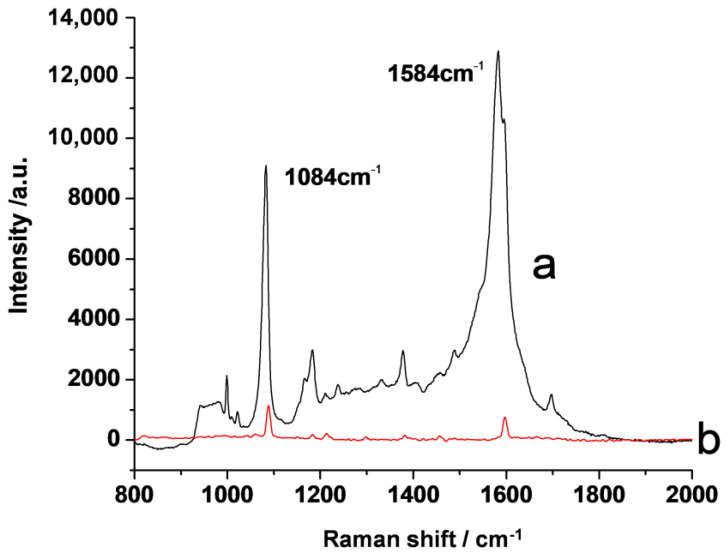
(**a**) SERS spectra of 4-MBT absorbed on the AgNP array-based substrate. The substrate was immersed in a 4-MBT ethanol solution (0.05 M) for ~2 h, and then it was picked out and rinsed with ethanol thoroughly before being tested; (**b**) SERS spectra of 4-MBT in ethanol solution. The concentration of 4-MBT is 0.1 M.

We employed 4-methylbenzenethiol (4-MBT) as the SERS probe molecules because of the strong Ag–S linkage. The enhancement factor (EF) was selected as a parameter for evaluating the enhancement of SERS bestowed upon it by the AgNP substrate. According to previous methods [40], the EF can be calculated using the following equation:

$$\mathrm{EF}=\frac{I_{sers}N_{bulk}}{I_{norm}N_{sers}}.$$

where *I_sers_* and *I_norm_* are the SERS and normal Raman intensities of the same peak, respectively, and *N_bulk_* and *N_sers_* are the numbers of probe molecules in the laser illumination volume in the bulk sample and on the SERS substrate. It should be noted that herein the peak area, rather than the peak intensity, is used to represent the SERS signal due to the significant different in the peak width [41]. We used the peak at 1584 cm^−1^ to estimate the EF because it was the strongest band in the Ramam spectra of 4-MBT. The peak at 1584 cm^−1^ can be assigned to the phenyl ring stretching motion (8a vibrational mode) [41]. The *I_sers_* and *I_norm_* values can be automatically calculated from the spectrum by our software (Figure 3). To determine *N_sers_*, we assumed that the coverage of the AgNPs on the silicon was 50 percent, which is a figure that we reached after analyzing the SEM images. The 4-MBT molecules with a molecular footprint of 0.19 nm^2^ were adsorbed as a complete monolayer on the half surface of the AgNPs. In our experimental condition, the laser spot area can be determined to be 1 µm^2^ and the penetration depth can be determined to be about 75 µm. Based on the above assumptions and the experimental results shown in Figure 3, the EF for the developed AgNP substrate was calculated to be 2.6 × 10^6^, which was high enough to enable sensitive SERS detection.

### 3.4. Application of the Proposed SERS-Active Substrate for MG Detection

**Figure 4:**
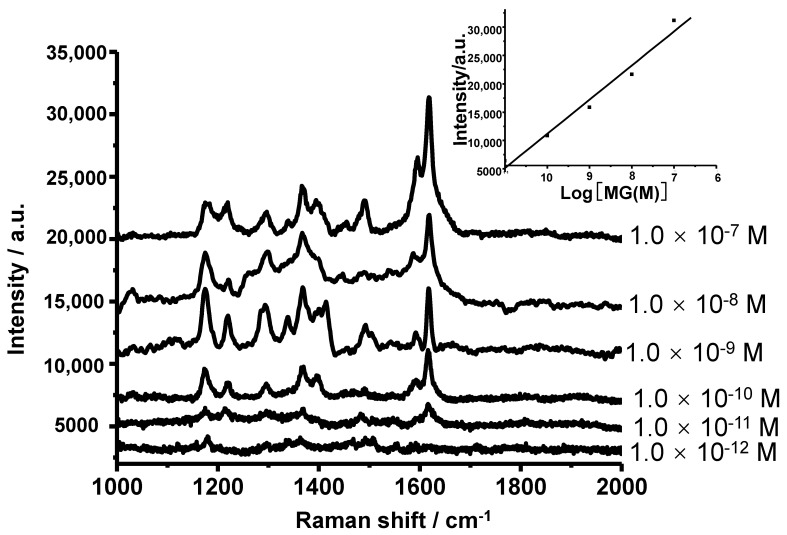
SERS spectra of MG at concentrations of 10^−12^, 10^−11^,10^−10^,10^−9^,10^−8^, and 10^−7^ M on the prepared substrate (*Insert*: the relationship between the peak intensity at 1620 cm^−1^ and the log value of the MG concentrations).

Ensuring the safety of seafood products entails a number of great challenges due to the risks of contamination with prohibited substances, such as crystal violet (CV) and malachite green (MG). Nowadays, however, there are only a few tentative applications of SERS detection in food safety tests. One of the biggest barriers to the application of SERS techniques is the lack of approaches for the preparation of economic, effective, and reliable SERS-active substrates. In this work, the utility of the AgNP array-based SERS substrate was demonstrated for the identification of MG. The substrates were immersed into MG solutions with concentrations ranging from 1.0 × 10^−7^ to 1.0 × 10^−12^ M for 2 h before undergoing Raman measurement. Figure 4 shows the Raman spectra of MG with different concentrations. The peaks of the different concentrations of MG were distinct and bright. The detection limit was determined to be 1.0 × 10^−11^ M, based on a signal to noise ratio greater than three. The strongest peak at 1620 cm^−1^ was selected to depict the relationship between MG concentrations and the corresponding peak intensity. There was a linear relationship between the intensity and the log value of MG concentrations ranging from 1.0 × 10^−11^ to 1.0 × 10^−7^ M.

**Figure 5:**
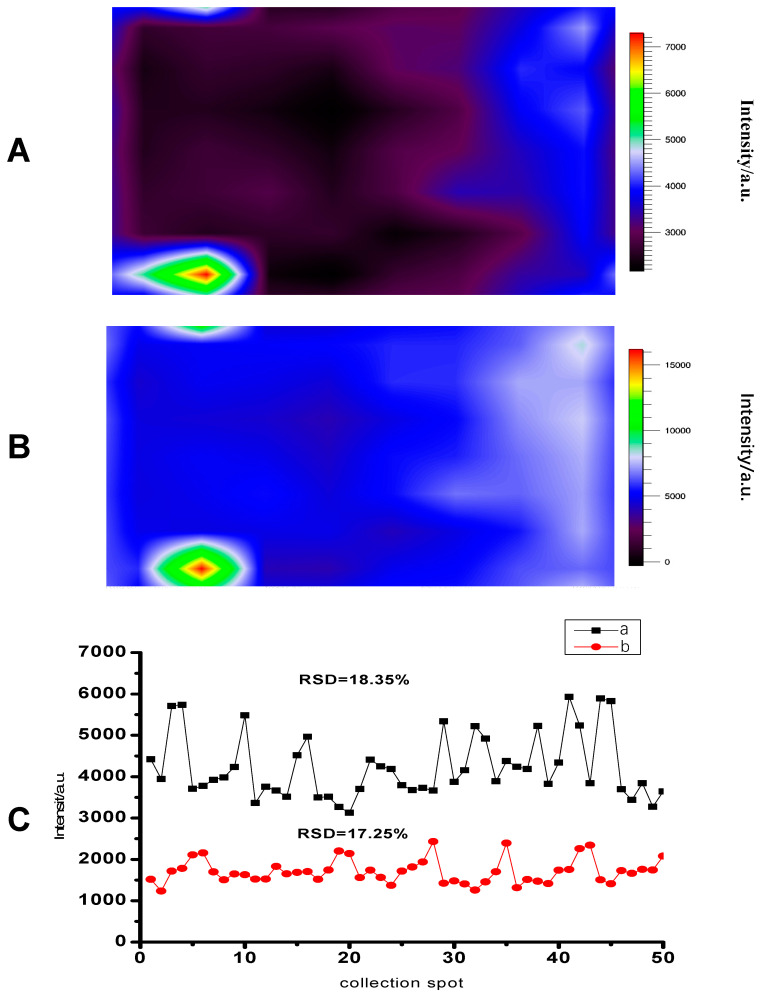
SERS mapping of the substrate using peaks of intensity at (**A**) 1368 cm^−1^ and (**B**) 1620 cm^−1^. (**C**) The peak intensities of MG SERS bands against the collection spots at (**a**) 1620 cm^−1^ and (**b**) 1368 cm^−1^. The concentration of MG was 1.0 × 10^−9^ M for the above tests. It should be noted that the much-enhanced intensity shown in the lower left corner of Figure 5A,B is a marker which we intentionally used to ensure that Figure 5A,B are captured at the same place. It could be a small cluster of AgNPs which shows strong SERS activity.

To evaluate the uniformity of the AgNP array-based SERS substrate, the SERS signal of fifty spots on the substrate were collected. Figure 5A,B show the typical mapping images of the 1368 cm^−1^ and 1620 cm^−1^ peaks of intensity of the MG solution on the substrate.

Black areas represent a lower intensity of the SERS signal. As seen from Figure 5A,B, the SERS peak area is homogeneous over the mapped area. Moreover, the standard deviation of the spectral intensity was less than 20% (Figure 5C), revealing the good uniformity and reproducibility of the developed SERS substrates.

## 4. Conclusions

In summary, we report a convenient strategy for the fabrication of highly uniform AgNP array-based SERS substrates at a large scale. CTAB was employed as the connection agent to guide the assembly of AgNPs on a silicon substrate. SEM images showed that the AgNPs were distributed on the modified silicon substrate with high density and lead to the creation of abundant “hot spots”. The SERS spectra of 4-MBT indicate that strong enhancement was achieved on the developed substrate with an EF of 2.6 × 10^6^ based on the Raman signals collected with a laser wavelength at 633 nm. To demonstrate the potential application of the proposed SERS-active substrate, we showed how the proposed substrate could be used for the detection of MG. The results showed that trace amounts of MG could be identified on the developed SERS substrate, and that a detection limit of 1 × 10^−11^ M was achieved. In view of the easy fabrication procedures and the highly uniform AgNP distribution, the proposed AgNP array-based SERS substrate holds great potential in the field of food safety testing.

## Data Availability

The data presented in this study are available on request from the corresponding author.
